# Retentive Force of Glass-Ceramic Soldered Customized Zirconia Abutment Copings with Prefabricated Titanium Bases

**DOI:** 10.3390/ma13143193

**Published:** 2020-07-17

**Authors:** Jeremias Hey, Monika Kasaliyska, Andreas Kiesow, Ramona Schweyen, Christin Arnold

**Affiliations:** 1 Department of Prosthodontics, Martin-Luther-University Halle-Wittenberg, Magdeburger Str. 16, 06112 Halle, Germany; jeremias.hey@uk-halle.de (J.H.); monika.burdinyashka@uk-halle.de (M.K.); christin.arnold@uk-halle.de (C.A.); 2 Fraunhofer Institute for Microstructure of Materials and Systems IMWS, Walter-Hülse-Str. 1, 06120 Halle, Germany; andreas.kiesow@imws.fraunhofer.de

**Keywords:** zirconia abutments, prefabricated titanium bases, glass solder, resin composite cement, retentive force, artificial aging

## Abstract

Two-piece abutments consisting of customized zirconia abutment copings and prefabricated titanium bases are popular due to their biological and esthetic advantages. Glass–ceramic solder (GS) is an alternative biocompatible connective agent. This in vitro study evaluated the retentive force of GS in comparison to classical resin composite cements (RC) after artificial aging and autoclaving. Ninety specimens consisting of prefabricated titanium bases and zirconia abutment copings were fabricated. The two parts of each specimen were fixed either by RC (n = 30) or GS with a luting space of either 30 µm (n = 30) or 100 µm (n = 30). Ten specimens of each group underwent autoclaving before artificial aging (water storage, thermocycling). Twenty specimens (including the 10 autoclaved specimens) of each group were exposed to a mechanical load. The retentive force between the zirconia and titanium in all specimens was determined. A fractographic analysis was performed to analyze the fracture surfaces of the GS specimens. The RC- and GS-connected two-piece abutments showed no relevant differences, independent of the luting space. RC appears to be more vulnerable to the thermal and mechanical loads than GS. Thus, GS may be an appropriate alternative to RC for two-piece abutments, especially for patients with enhanced biocompatibility requirements.

## 1. Introduction

In dentistry, the fabrication of fixed partial dentures using dental implants became popular during the last decades [1]. For this reason, dental implants have been used to replace lost tooth roots. Abutments connect implants with artificial crowns. Conventionally, abutments are made of titanium. The connection between the implant and the abutment is a critical area. Here, high biological compatibility and high stability are required [2]. For esthetic reasons, the material used should be tooth-shaded. Up to now there is no material that fulfils all requirements. Therefore, compound materials are used. Zirconia abutments have recently become popular due to their biologic and esthetic advantages. Compared to titanium, zirconia shows lower plaque retention because of its smooth surface, making it especially suitable for use in the esthetic zone [2,3]. However, the fracture strength of zirconia is lower than that of titanium [4]. The development of customized zirconia abutment copings with prefabricated titanium bases was intended to combine the mechanical properties of titanium with the esthetic and biologic advantages of zirconia [5,6,7]. Although the fracture strength of these two-piece abutments has been proven to be higher than that of pure zirconia abutments, an optimal connection between the two materials is essential to guarantee long-term stability [5,6,7,8,9,10]. This connection is usually realized with resin composite cements. However, an alternative concept using glass–ceramic solder for bonding titanium and zirconia was patented in 2012 [11].

The connection of the zirconia abutment copings and titanium bases using glass–ceramic solder yields two interfaces with distinct adhesion mechanisms. The adhesion mechanism between titanium and glass–ceramic solder is similar to that in a classic metal-silicate ceramic composite. The mechanical retention is primarily decisive for the adhesive bond. The ceramic mass, which should have a lower coefficient of thermal expansion (CTE) than the metal alloy, shrinks onto the metal surface during the cooling process. In addition, adhesive oxides are formed during the heating of the metal. These can form a chemical bond with silicon dioxide. This bonding technology is considered mature and is recommended for routine clinical use [12,13,14,15]. In contrast, the bond between the glass–ceramic solder and zirconia is based on mechanical adhesion. Unlike the metal-silicate ceramic bond, the chemical bond between the zirconia structure and the veneer is not based on adhesive oxides but is rather dependent on hydrogen bridge bonds. This bond is described as good, but the adhesion values do not reach the same dimensions as those of the classic metal-silicate ceramic bond [16,17,18,19].

To date, only two studies from the field of dental medicine have dealt with the glass–ceramic solder bond between zirconia and titanium. In a four-point bending test, Mick et al. determined strength values of 104–119 MPa between zirconia and titanium cylinders joined with glass–ceramic solder. On average, these values were five times higher than those of a control group luted with composite (13–30 MPa) [20]. In the other study, Vu et al. observed up to twice as high bond forces in the glass solder group (14–17 MPa) compared to the control group luted with a composite (8 MPa) in shear tests of zirconia and titanium cylinders joined with glass–ceramic solder [21]. Glass solder bonds have the advantage of being able to be hermetically sealed, thereby preventing microbial penetration. They can also withstand higher temperatures. These properties appear to be an advantage for the bonding of two-piece abutments, especially when they have to be sterilized. Although the necessity of sterilizing two-piece abutments is still debatable, it is in any case obligatory in the one-time concept [22].

Currently, there are no data describing the stability of the bond between zirconia abutment copings and prefabricated titanium bases joined with glass solder in comparison with conventional luting with resin composite cements. This study aims to investigate the retentive force between zirconia copings and titanium bases luted with resin composite cements or glass solder, after autoclaving, artificial aging, and mechanical loading. The glass solder specimens in this study are also subjected to a fractographic analysis. The following hypotheses were tested: (1) The connection of customized zirconia abutment copings with prefabricated titanium bases using glass–ceramic solder enhances the retentive force in comparison to resin composite cement; (2) neither autoclaving nor mechanical loading affects the retentive force of resin composite cements or glass solder; and (3) an enlargement of the joining gap in specimens joined with glass solder does not increase the retentive forces.

## 2. Materials and Methods

**Figure 1:**
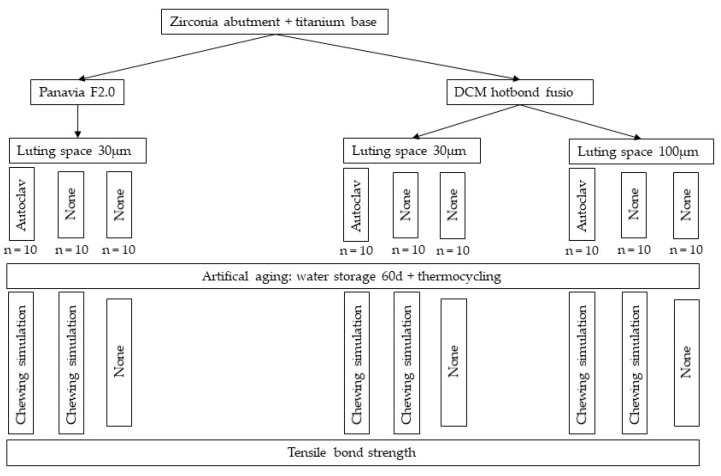
Study protocol.

The study protocol is presented in Figure 1.

### 2.1. Preparation of 11 Specimens

The materials used in the study, their composition, and manufacturers are listed in Table 1.

**Figure 2:**
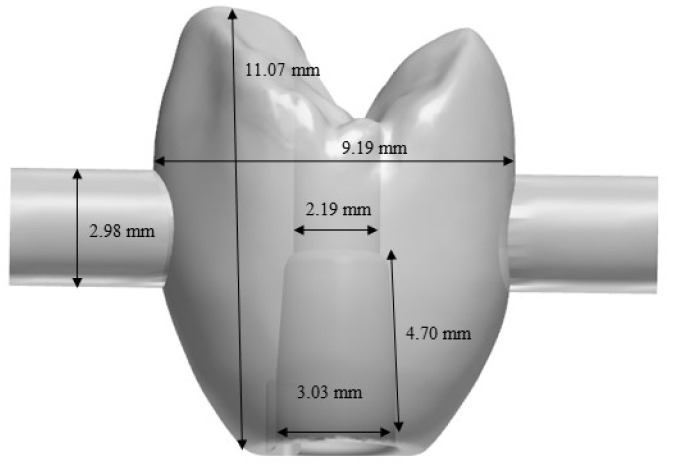
Dimensions of the zirconia abutment coping fabricated by computer-aided design/computer-aided manufacturing (CAD-CAM).

To enable mechanical loading in the chewing simulator, zirconia abutment copings in the shape of a premolar crown were designed using computer-aided design (CAD) software (3Shape dental system 2.15.4.0, 3ShapeA/S, Copenhagen, Denmark). Two cylindrical retention aids were added to the vestibular and oral surfaces in the area of the crown equator for the planned pull-off tests. In the 2-D cross-section, the zirconia abutment copings exhibited the geometry shown in Figure 2. For 60 zirconia abutment copings, the luting space was set to 30 µm, and for 30 zirconia abutment copings, the space was set to 100 µm. From a zirconia blank (Organic Zirkon translucent, Organical CAD/CAM GmbH, Berlin, Germany), the zirconia abutment copings were milled out under air cooling on a 5-axis milling machine (Organical 5X, Organical CAD/CAM GmbH, Berlin, Germany) and then sintered at 1450 °C for 2 h in a furnace (Organical Heat S, Organical CAD/CAM GmbH, Berlin, Germany).

### 2.2. Luting the Components with Resin Composite Cement

A total of 30 zirconia abutment copings with a luting space of 30 µm were luted with resin composite cement (group RC30). For this purpose, the titanium bases were screwed onto implant analogs (K 3010.4300, Camlog Biotechnologies AG, Basel, Switzerland) to protect the interface. The shoulders of the titanium bases and screw channel openings were covered with adhesive wax to protect them from blasting sand. The luting surfaces of both the titanium bases and the zirconia abutment copings were blasted with 50 µm aluminum oxide and a pressure of 1.5 bar. Subsequently, the components were thoroughly cleaned with oil- and water-free compressed air. The bonding surfaces were conditioned with primers for at least one minute, namely the titanium bases with Alloy Primer (Kuraray Noritake Dental Inc., Okayama, Japan) and the zirconia abutment copings with Clearfil Ceramic Primer Plus (Kuraray Noritake Dental Inc., Osaka, Japan). Pastes A and B of the luting composite (Panavia F 2.0, Kuraray Noritake Dental Inc., Osaka, Japan) were then mixed in a ratio of 1:1 and applied to the surfaces to be luted. After joining the components, the excess was removed, and an oxygen inhibitor (Oxyguard II, Kuraray Noritake Dental Inc., Osaka, Japan) was applied in the screw channel and at the luting space. After a setting time of 20 min, the specimens were finished and polished.

### 2.3. Joining the Components with Glass Solder

A total of 30 zirconia abutment copings with a luting space of 30 µm (group GS30) and 30 zirconia abutment copings with a luting space of 100 µm (group GS100) were luted with glass solder. The titanium bases were screwed onto the laboratory analogs and covered with adhesive wax in the shoulder areas. The luting surfaces of both the titanium bases and the zirconia abutment copings were blasted with 50 µm aluminum oxide at a pressure of 1.5 bar. Subsequently, the components were thoroughly cleaned with oil- and water-free compressed air. Afterwards, the screw channel openings were sealed with a firing pillow (DCM hotbond fix, Dental Creativ Management, Rostock, Germany) to protect them against solder run-in. The titanium surfaces underwent a coating with a connect spray (DCMhotbond fusio connect spray, Dental Creativ Management, Rostock, Germany) and a sintering process. The temperature was increased at a rate of 55 K/min under vacuum up to a value of 800 °C and was maintained for 1 min in a ceramic firing furnace (Organical Heat HT, Organical CAD/CAM GmbH, Berlin, Germany). The powdery base material of the glass solder (DCMhotbond fusio 12, Dental Creativ Management, Rostock, Germany) that was dissolved in an alcoholic fluid (DCMhotbond fusio liquid, Dental Creativ Management, Rostock, Germany) was sprayed onto the preconditioned surfaces. For sintering, the compound specimens were thermally treated in the abovementioned furnace. The temperature was increased to a rate of 40 K/min under vacuum up to a value of 770 °C and was maintained for 1 min. After sintering and a slow cooling process (1 K/min), the specimens were cleaned by steaming. Finally, the excess solder was removed, and the edges were finished and polished.

### 2.4. Specimen Pretreatment

First, 10 specimens from each group (./. autoclave) were autoclaved three times at 134 °C and a pressure of 3.1 bar for 3 min (DAC Universal, Sirona Dental Systems GmbH, Bensheim, Germany). Then, all specimens were artificially aged. They were stored in water at 37 °C for 60 days in an incubator (BE 500, Memmert GmbH & Co. KG, Schwabach, Germany) and then subjected to an additional 30,000 thermal load changes in a thermal cycler with hot and cold baths (WEDC1V, version 2.5, Willytec, SD Mechatronik GmbH, Feldkirchen-Westerham, Germany). Per cycle, the specimens remained in the water bath for 30 s (Aqua dest.) at temperatures of 5 and 55 °C [5]. The dripping time of 5 s in between prevented mixing of the two water baths.

The 10 autoclaved specimens of each group (./. autoclav) as well as 10 additional specimens (./. chew) from each group were exposed to additional mechanical stress. A four-chamber, two-axis Willytec chewing simulator (CS-4.4, SD Mechatronik GmbH, Feldkirchen-Westerham, Germany) was used for this purpose. Implant analogs were fixed in holding devices made for this purpose, and the specimens were screwed into these. Aluminum oxide spheres (precision sphere F 610-84500-0, Friatec AG, Mannheim, Germany) with 5-mm diameter were used as an antagonist. The spheres were polymerized with a cold-curing polymer (Pala X Press, Heraeus Kulzer, Hanau, Germany) in individual antagonist sockets in the chewing simulator. They were placed in such a way that one contact point each was made outside the screw channel opening on the vestibular and oral cusp slopes. Each of the 60 specimens to be aged was then subjected to 1.2 million load cycles (vertical stroke, 2 mm; lateral stroke, 0.7 mm; vertical and lateral stroke speed, 55 mm/s) with an average functional masticatory force of 50 N [24,25]. This corresponds to a wearing period of five years. The mechanical loads were applied in a saliva bath (Glandosane: Aqua dest. 1:2, Cell Pharm GmbH, Bad Vilbel, Germany) at room temperature.

The 10 remaining specimens of each group (./. none) were not subjected to any measures other than aging.

### 2.5. Determination of the Retentive Force

The retention between the zirconia copings and the titanium bases was measured in tension by using a universal testing machine (Zwick Z010/TN2A, ZwickRoell, Ulm, Germany) at a crosshead speed of 2 mm/min. The specimens were screwed to the lab analogs. The specimens were then inserted into the towing device, with the lab analog being screwed tightly in the lower holding device and the zirconia abutment coping subsequently being placed into the upper holding device, which was fixed moment-free to the testing machine with one chain. The forces and loads (tensile bond load until debonding [N/ bonding area 50.3 mm^2^ = MPa]) that were required to separate the zirconia copings from the titanium inserts were recorded. The data were collected using the program testXpert II (ZwickRoell Ulm, Germany).

### 2.6. Fractographic Analysis

To analyze the glass solder bond between titanium and zirconia, the fracture surfaces of three titanium bases from the GS100.autoclave group joined with glass solder were recorded by means of scanning electron microscopy (SEM, Supra 55 VP; Carl Zeiss Microscopy, Jena, Germany) and energy-dispersive X-ray spectroscopy (EDX, Octane Elite, EDAX, Mahwah, NJ, USA) to assess their elemental composition. Due to its size and rotationally symmetrical shape, the fracture surface of the titanium bases was divided into six measurement positions. One EDX spectrum was recorded for each position. A map was created for the chemical elements titanium (Ti), silicon (Si), and zirconium (Zr). Based on element mapping, a quantitative analysis was then performed (cellF, Version 2.6 (Build 1200), Olympus Soft Imaging Solutions, Münster, Germany). The degree of the coverage or the percentage of the surface area on the titanium base was determined.

### 2.7. Statistical Analysis

After descriptive analyses, results were tested with respect to normal distribution using a Kolmogorov–Smirnov test. Since the results were normally distributed, *t*-tests, one-way analysis of variance (ANOVA), and Bonferroni tests were used to analyze the differences between the groups. *P* values lower than 0.01 were considered to be statistically significant. The analysis was performed using IBM SPSS Statistics, version 23.0 (IBM Inc., Ehningen, Germany).

## 3. Results

One specimen from the group RC.none and one specimen from the group GS100.chew showed an incorrect bond. Their retentive forces were below 10 N. Both specimens were not considered in the statistical evaluation. Table 2 summarizes the calculated mean values and standard deviations as well as the minimum and maximum measured retentive forces of the individual groups.

**Figure 3:**
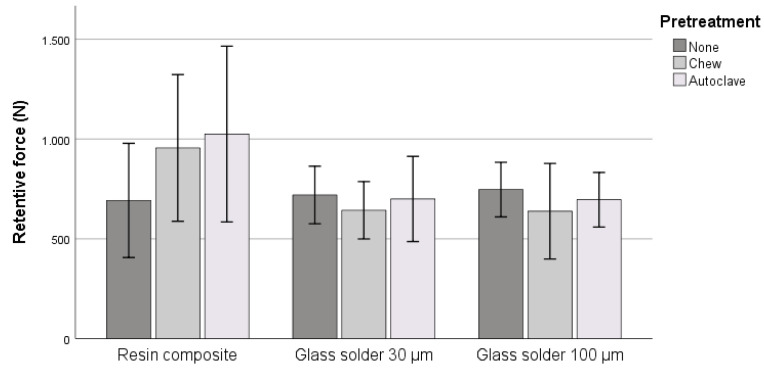
Retentive forces of the three groups after different pretreatment protocols.

For the specimens that were only aged (group.none), the lowest mean retentive forces were observed for RC.none (692.33 ± 285.97 N), and the highest values were observed for GS100.none (746.70 ± 136.77 N, see Figure 3). After mechanical loading, the retentive forces in the group RC.chew were higher by an average of 37%. This was accompanied by an increased dispersion of the measured values (*p* = 0.41). A 10% reduction in the mean pull-off forces (*p* = 0.97) was noted between GS30.none and GS30.chew, while a 15% reduction in the mean pull-off forces (*p* = 0.57) was observed between GS100.none and GS100.chew. The RC.chew specimens luted with composite showed higher retentive forces than the glass solder-joined specimens GS30.chew and GS100.chew (39%, *p* = 0.05).

Among the initially autoclaved specimens, the luted RC.autoclav showed higher pull-off forces on average than the soldered GS30.autoclav (31%, *p* = 0.158) and GS100.autoclav (32%, *p* = 0.131). Furthermore, there was a high standard deviation among the luted specimens (see Table 2). Compared to group RC none, which had only aged, the mean forces had increased by 48% (*p* = 0.19).

Between the glass solder groups GS30.autoclav and GS30.none, a 3% reduction in the mean pull-off forces was observed (*p* = 1). Between the glass solder groups GS100.autoclav and GS100.none, a 7% reduction in mean pull-off forces was noted (*p* = 1).

### Surface Analysis by Scanning Electron Microscopy and Energy-Dispersive X-Ray Spectroscopy

Table 3 shows an example of the quantitative composition of a section of the fracture surface of a titanium base from group GS100.autoclave determined by energy-dispersive X-ray spectroscopy (EDX).

**Figure 4:**
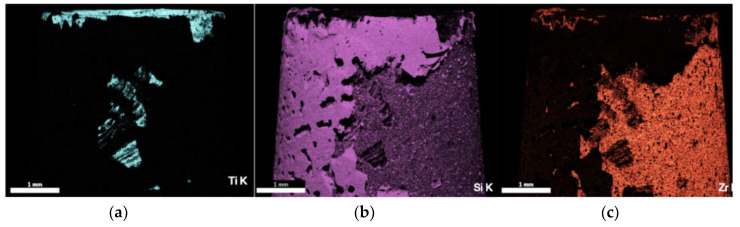
Distribution of the elements titanium (**a**), silicon (**b**), and zirconium (**c**) by energy-dispersive X-ray spectroscopy (EDX)-mapping. Bright areas indicate higher element concentrations.

Remarkably, zinc was detected, even though the element is not present in any of the starting materials, according to the manufacturer. The fracture surfaces consistently showed higher proportions of zirconium and silicon compared to titanium. The concentrations of the elements titanium (blue-gray), silicon (violet), and zirconium (orange) in a specimen section are shown as an example in Figure 4.

High concentrations appear intensely bright. In the black areas, the element is below the detection limit (approx. 0.5%). High concentrations of titanium are found (Figure 4a) in a small area in the center and on the edge of the titanium base. Large areas of the fracture surface are covered by silicon (Figure 4b) and zirconium (Figure 4c). Table 4 summarizes the distribution of the elements titanium, silicon, and zirconium on the examined fracture surfaces of the three titanium bases from the group GS100.autoclav.

**Figure 5:**
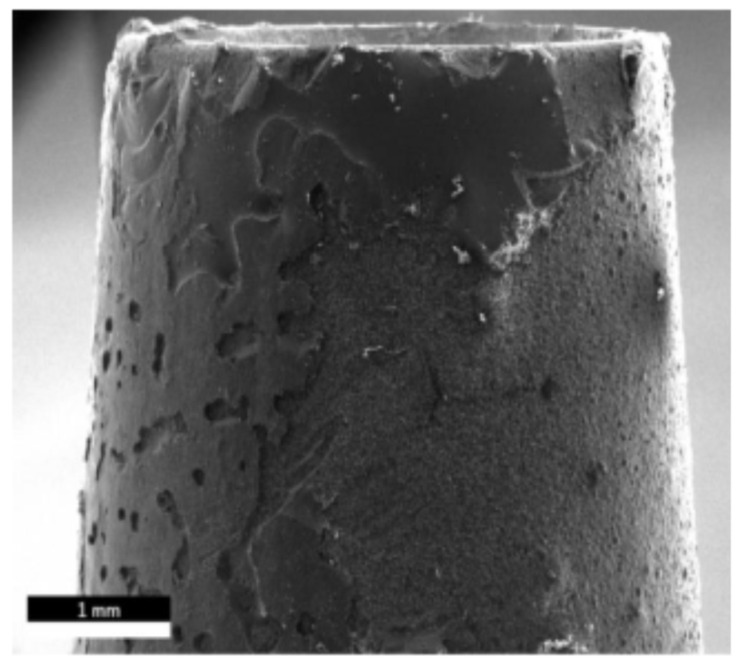
Corresponding to the EDX analysis shown in Figure 4, this figure shows the scanning electron microscopy (SEM) analysis of the same specimen.

The exposed proportion of titanium on the fracture surface is very low, averaging 3.75%. The silicon content varies between 31% and 74% (mean: 50.9%), and the zirconium content is between 11.3% and 60% (mean: 38.8%). Figure 5 shows the SEM image corresponding to Figure 4.

In addition to pore formation, a mixed adhesive–cohesive fracture between the glass solder and the zirconia is visible. Based on the findings obtained from the SEM image and the EDX analysis, the group GS100.autoclav can be concluded to have a fracture in the area of the boundary layer between the glass solder and the zirconia abutment coping.

## 4. Discussion

The present study investigated the retentive force between zirconia copings and titanium bases luted with resin composite cements or glass solder after autoclaving, artificial aging, and mechanical loading. Specimens that underwent aging showed no relevant differences between the two bonding methods. Mechanical loading and sterilization led to an increase in the retentive forces with a high standard deviation in the luted specimens. The applied mechanical loads and sterilization did not reduce the retentive forces of the soldered specimens. Moreover, an enlargement of the solder gap had no influence on the retentive forces in the specimens joined with glass solder. The EDX analysis indicates a fracture between the glass solder and the zirconia.

For the resin composite-cemented specimens, an average retentive force of 692.33 N was measured after artificial aging (water storage for 60 days and 30,000 cycles of temperature cycling). Studies with comparable pretreatment showed similar values. Mehl et al. found retentive forces of 687.7 N, while Arce et al. found 595.5 N and Gehrke et al. at 532.4 N [6,26,27].

In accordance with the findings reported by Zenthöfer et al., an average increase in adhesive strength was observed after artificial aging (60 days water storage and 35,000 cycles of temperature cycling) and chewing simulation (1.2 million cycles) in the group RC.chew (*p* = 0.410) [28]. However, the specimens in the present study showed a strong standard deviation. As in the study presented by Al-Sheri et al., microcracks could have occurred as a result of mechanical stress in some specimens, which exerted a negative influence on the adhesive bond [29].

In accordance with the results reported by Fadanelli et al., a significant average increase in adhesive strength was observed after initial autoclaving (3× 134 °C), artificial aging (water storage for 60 days and 35,000 cycles of temperature cycling), and chewing simulation (1.2 million cycles) in the group RC.autoclav (*p* = 0.19) [30]. One reason for this could be the increase in water absorption of the luting composite with rising temperatures, which is accompanied by swelling. However, the possibility of further polymerization caused by the application of heat and the greater stable cross-linking as a result is also being discussed [31].

To our knowledge, there are only two studies to date that have dealt with the joining of titanium and zirconia using glass solder. Mick et al. investigated the bending strength of the glass solder bond between titanium and zirconia [20]. For this purpose, titanium and zirconia cylinders with diameters of 10 mm were fabricated and sandblasted on the base and top surfaces. A part of the specimens was thus joined with glass solder at the conditioned connecting surfaces, while the rest was adhesively luted with composite. After water storage for 30 or 90 days and heat and pressure application for 14 days at 70 °C and 4.9 bar, the bending strength of the bond was determined in a four-point bending test. Here, the glass solder bond showed strength values of 104 to 119 MPa, which were up to six times higher than the values in the group of luted specimens, which were between 13 and 30 MPa.

In the second study, Vu et al. tested the shear strength of the glass solder joint of titanium and zirconia in a different test setup [21]. For this purpose, they used titanium and zirconia cylinders with 5-mm diameters for the base and top surface. After sandblasting these connecting surfaces, the specimens were divided into groups. In the control group, the specimens were adhesively luted with composite, while in the remaining groups, a joint was made with different compositions of the glass solder. At 91%, the glass solder consisted of SiO_2_, B_2_O_3_, Al_2_O_3_, Na_2_O, and K_2_O. The effects of the addition of 1 wt.% Fe_2_O_3_ and of 1 wt.% Fe_2_O_3_ with 1 wt.% Bi_2_O_3_ respectively was investigated. During the shear strength test in a universal testing machine, shear strength values of 14 to 17 MPa were determined in the groups of the soldered specimens.

In our study, the tensile bond strength for the specimens joined by glass solder was 12.7–14.8 MPa. Notably, the two previous studies and the present study used different test methods and specimens with different geometries. Due to the closed ring structure of the luting bases in the present study, higher forces would be expected than with the joined cylinders in the study by Vu et al. [21]. The chewing simulation did not show any influence on the retentive forces. None of the specimens failed under the mechanical load. An expected structural weakening of the ceramic glass solder mass due to mechanical fatigue was not observed. After autoclave sterilization at 134 °C, no impairment of the glass solder bond could be detected either. Similarly, Mick et al. could not detect any negative influence of aging at 70 °C below 4.9 bar on the specimens joined with glass solder [20].

A space of 30 µm has become established for adhesive abutments [5,7]. In a study by Ebert et al., an adhesive space of 30 µm showed a statistically significant influence on the increase in retention compared to an adhesive gap of 60 µm. Mehl et al. compared the retention forces at an adhesive space of 60 and 100 µm and observed a better bond at an adhesive space of 60 µm [27]. When soldering with DCM hotbond, however, the manufacturer points out that a solder space below 100 µm and above 300 µm is contraindicated. Nevertheless, in half of the samples joined with glass solder, the 30 µm solder space recommended for composites was used either to quantify the bond when the space width fell below the required width or to enable a comparison independent of the space width. For the other half, a required minimum space width of 100 µm was observed.

However, in the present study, the width of the solder gap showed no relevant influence on the retentive forces, independent of autoclaving, artificial aging, or mechanical loading.

The pore formation observed in the SEM images (cf. Figure 5) in the specimens joined with glass solder in the present study could be due to the residual moisture of the glass solder components that evaporated during sintering. According to Hong et al., they could also be the result of a reaction between titanium and silicon dioxide [32] (1):

5Ti + 3SiO_2_→Ti_5_Si_3_ + O_2_↑ (1)

In order to alleviate this problem and improve the bond between titanium and glass–ceramic, cobalt oxide can be added to the glass–ceramic mass. This causes the following reaction (2).

Ti + 2CoO→2Co (precipitate) + TiO_2_ (2)

The additional wedging of the cobalt precipitate resulted in an improvement of the adhesive forces in experiments [33].

The surface analysis of the titanium bases in the present study revealed a material failure, primarily along the zirconia-glass solder interface. This is not consistent with previous publications. For the evaluation of the fracture surfaces, Mick et al. used SEM and EDX, while Vu et al. used light microscopy with 25× magnification [20,21]. In both studies, the bond most frequently failed cohesively in the glass solder or in the area of the glass solder-titanium bond. The reasons for these different results could be related to the differences in the test setup and the specimen geometry.

The bond between titanium and glass solder can be improved by modifying the composition of the glass solder. Brow et al. considered the reaction product of titanium and silicon dioxide, titanium silicide (Ti_5_Si_3_), to be a weak point of the glass solder bond [34]. An alternative material would be a boroaluminate-based glass solder. Its use does not produce a gaseous product as in the case of glass solder containing silicon [35]. However, the effects of the application of this glass solder in humans are not yet known. In clinical studies, the bond between zirconia and glass–ceramic proved to be more problematic than the bond between glass–ceramic and metal [36]. One reason is assumed to be the phase transformation of the material during veneering. Due to the heat generated during sandblasting, the CTE can change from approx. 10.5−10^−6^/K in the tetragonal phase to approx. 7−10^−6^/K in the monoclinic phase. However, the CTE of the glass solder is aligned with the tetragonal phase. Thus, adhesion-reducing occurs in monoclinic areas. This phase transformation can be reversed by heat treatment after sandblasting [17]. However, the positive effect of this measure is not guaranteed [37]. In order to prevent potential surface changes of the zirconia ceramic due to aggressive sandblasting, the specimens in the present study were sandblasted with 50 µm Al_2_O_3_ at a pressure of 1.5 bar.

In conclusion, the use of glass solder to join titanium bases and zirconia abutments yielded retentive forces that are equivalent to those achieved with composite luting. To what extent these in vitro results are also applicable to everyday clinical practice cannot be clearly stated to date. In practice, many more factors, such as the ease of processing, production time, cost-effectiveness, acceptance by dentists and dental technicians, would influence the bonding method that will ultimately be used for two-piece abutments. In this regard, further studies—especially clinical studies—with the glass solder material should be conducted before a final recommendation for the procedure can be made. Two relevant aspects of the clinical application can be derived from the study. Firstly, the glass solder bond achieves and retains bond strengths—even after aging—that are of the same order of magnitude as those achieved with clinically established types of composite cement. Secondly, the glass solder composite tolerates the clinically necessary autoclave sterilization procedures without a relevant influence on the bond strength. Based on this knowledge, clinical investigations can now be conducted on the behavior of the composite concerning bacterial colonization and inflammatory reactions of the peri-implant tissue.

Furthermore, autoclave sterilization did not show any negative effects on the luted abutments. On the basis of the present study, it can therefore be recommended that this form of sterilization can be used in the future, especially for procedures involving blood contact—such as the one-abutment-one-time concept—without restrictions for the adhesive bond.

## Figures and Tables

**Table 1:** Materials, compositions, and manufactures.

Materials	Composition	Manufacturer
CAD/CAM titanium base	Titanium 90 wt.%, aluminum 6 wt.%, vanadium 4 wt.%, ferrum ≤ 0.25 wt.%, carboneum ≤ 0.08 wt.%, nitrogen ≤ 0.05 wt.%, oxygen ≤ 0.13 wt.%, hydrogen ≤ 0.012 wt.%	CAMLOG Biotechnologies, Basel, Switzerland
Zirconia abutment coping	3Y-TZP ZrO_2_ + HfO_2_Y_2_O_3_ > 99.0 wt.% (Y_2_O_3_ 5.15 +/− 0.20 wt.%; HfO_2_ < 5.0 wt.%); Al_2_O_3_ < 0,25 +/− 0.10 wt.%; Fe_2_O_3_ < 0.1 wt.%; Na_2_O < 0.04 wt.%	Organic Zirkon, R&K CAD/CAM Technologie, Berlin, Germany
Lab analog	Titanium 90 wt.%, aluminum 6 wt.%, vanadium 4 wt.%	CAMLOG Biotechnologies, Basel, Switzerland
Panavia F 2.0	Monomer matrix: 10-MDP, hydrophobic aromatic and aliphatic, photoinitiator, dibenzoyl peroxide dimethacrylate, hydrophilic dimethacrylate, sodium aromatic sulphinate, N,N-diethanol-p-toluidine; inorganic fillers: silanated silica, barium glass, colloidal silica; initiators, accelerators; catalysators; camphorquinone; pigments; sodium fluoride	Kuraray Noritake Dental, Osaka, Japan
Alloy primer	Acetone 6-(4-vinylbenzyl-N-propyl)amino-1,3,5-triazine-2,4-dithione, 10-MDP	Kuraray Noritake Dental, Osaka, Japan
Clearfil Ceramic Primer Plus	Ethanol, 3-methacryloxypropyl trimethoxy silane, 10-MDP ^1^	Kuraray Noritake Dental, Osaka, Japan
Panavia F 2.0 Oxyguard II	Glycerin, polyethylene, glycol, catalysators, initiators, pigments	Kuraray Noritake Dental, Osaka, Japan
Glass solder ^2^	SiO_2_ 60–70wt.%, Al_2_O_3_ 4–10 wt.%, K_2_O 6–10 wt.%, Na_2_O 6–10 wt.%	Dental Creativ Management, Rostock, Germany
Connect spray ^3^	SiO_2_, Al_2_O_3_, K_2_O, Na_2_O, CaO, B_2_O_3_	Dental Creativ Management, Rostock, Germany

^1^ 10-methacryloyloxydecyl dihydrogen phosphate. ^2^ Composition according to Mick et al. (2013) [23]. ^3^ Percentage distribution of the ingredients is not accessible.

**Table 2:** Descriptive statistics of the retentive forces (N) and tensile bond strengths (MPa).

Test Group		n	Retentive Force (N)			Tensile Bond Strength (MPa)
			Mean ± SD	Min.	Max.	Mean
Resin composite cement	RC.none	9	692.33 ± 285.97	426	1310	13.8
	RC.chew	10	955.30 ± 367.22	293	1450	19
	RC.autoclav	10	1024.90 ± 440.28	403	1440	20.4
Glass solder 30 µm	GS30.none	10	719.70 ± 144.21	526	956	14.3
	GS30.chew	10	643.10 ± 143.69	300	782	12.8
	GS30.autoclav	10	699.70 ± 213.31	258	906	13.9
Glass solder 100 µm	GS100.none	10	746.70 ± 136.77	530	947	14.8
	GS100.chew	9	638.33 ± 239.39	185	1030	12.7
	GS100.autoclav	10	695.80 ± 136.89	453	929	13.8

**Table 3:** Element spectrum determined by energy-dispersive X-ray spectroscopy (EDX).

Element	% by Weight	% by Atom	Error %	Element	% by Weight	% by Atom	Error %
C	4.0	8.7	7.0	Zr	14.7	4.2	1.5
Si	23.7.0	21.7	2.0	Cl	0.3	0.2	4.8
O	26.4	42.5	5.7	K	7.8	5.1	2.0
Zn	2.1	0.8	5.3	Ca	1.7	1.1	2.7
Na	4.3	4.9	3.3	Ti	8.5	4.6	2.0
Al	6.4	6.2	2.2	Zn	2.1	0.8	5.3

**Table 4:** Quantitative determination of the elements titanium (Ti), silicon (Si), and zirconium (Zi).

Trial Sample	Ti			Si			Zr		
	Mean ± SD	Min.	Max.	Mean ± SD	Min.	Max.	MW ± SD	Min.	Max.
P1	1.9 ± 2.3	0.04	5.2	44.9 ± 12.8	31.1	62.8	41.9 ± 13.7	23.3	59.6
P2	6.4 ± 10.2	0.09	26.8	50.6 ± 13.05	38.8	73.8	40.2 ± 16.3	16.6	57.6
P3	2.9 ± 3.8	0.02	*10.1*	57.2 ± 7.8	49.4	71.2	34.5 ± 17.5	11.3	52.6
Mean	3.8			50.9			38.8		

P, specimen; Min., minimum; Max., maximum; SD, standard deviation.
